# Cerebral Dynamics during the Observation of Point-Light Displays Depicting Postural Adjustments

**DOI:** 10.3389/fnhum.2017.00217

**Published:** 2017-05-08

**Authors:** Eduardo F. Martins, Thiago Lemos, Ghislain Saunier, Thierry Pozzo, Daniel Fraiman, Claudia D. Vargas

**Affiliations:** ^1^Laboratório de Neurobiologia II, Instituto de Biofísica Carlos Chagas Filho, Universidade Federal do Rio de JaneiroRio de Janeiro, Brasil; ^2^Programa de Pós-Graduação em Ciências da Reabilitação, Centro Universitário Augusto Motta—Centro Universitário Augusto Motta (UNISUAM)Rio de Janeiro, Brasil; ^3^Laboratório de Cognição Motora, Departamento de Anatomia, Universidade Federal do ParáPará, Brasil; ^4^Institut National de la Santé et de la Recherche Médicale-U1093 Cognition, Action, et Plasticité Sensorimotrice, UFR STAPS, Université de BourgogneDijon, France; ^5^Laboratorio de Investigación en Neurociencia, Departamento de Matemática y Ciencias, Universidad de San AndrésBuenos Aires, Argentina; ^6^Consejo Nacional de Investigaciones Científicas y Técnicas (CONICET)Buenos Aires, Argentina

**Keywords:** electroencephalography, superior temporal sulcus, point-light display, action observation, balance

## Abstract

**Objective:** As highly social creatures, human beings rely part of their skills of identifying, interpreting, and predicting the actions of others on the ability of perceiving biological motion. In the present study, we aim to investigate the electroencephalographic (EEG) cerebral dynamics involved in the coding of postural control and examine whether upright stance would be codified through the activation of the temporal-parietal cortical network classically enrolled in the coding of biological motion.

**Design:** We registered the EEG activity of 12 volunteers while they passively watched point light displays (PLD) depicting quiet stable (QB) and an unstable (UB) postural situations and their respective scrambled controls (QS and US). In a pretest, 13 volunteers evaluated the level of stability of our two biological stimuli through a stability scale.

**Results:** Contrasting QB vs. QS revealed a typical ERP difference in the right temporal-parietal region at an early 200–300 ms time window. Furthermore, when contrasting the two biological postural conditions, UB vs. QB, we found a higher positivity in the 400–600 ms time window for the UB condition in central-parietal electrodes, lateralized to the right hemisphere.

**Conclusions:** These results suggest that PLDs depicting postural adjustments are coded in the brain as biological motion, and that their viewing recruit similar networks with those engaged in postural stability control. Additionally, higher order cognitive processes appear to be engaged in the identification of the postural instability level. Disentangling the EEG dynamics during the observation of postural adjustments could be very useful for further understanding the neural mechanisms underlying postural control.

## Introduction

Sculpted by natural selection through hundreds of millions of years of evolution, the motor system acts as a mediator between an animal and its environment, playing an important role in its survival and interactions with other animals, including challenges like searching for food, self-defense, and courtship (Nudo and Frost, 2009). For these reasons, the observation and understanding of others' actions have been considered crucial for animals' social interaction and survival (Iacoboni and Dapretto, 2006). Through action observation, animals not only have the ability to learn from others and react to their actions, but also to anticipate and infer from them (Iacoboni and Dapretto, 2006), acquiring more refined and efficient motor repertoires.

The understanding of the cerebral dynamics taking place during the observation of other's actions has advanced remarkably with the use of paradigms employing the recognition of biological motion (BM). This approach called point-light display (PLD) employs the observation of a dozen light dots attached on the main joints of an actor generating a motion display characterizing the kinematics of movements such as jumping, running, or paddling, among others (Johansson, 1973, 1976). These PLD animations permit the observation of the kinematic features of motion—without distractors like color and texture—and evoke a vivid percept of human actions (Johansson, 1973, 1976). When contrasted with “scrambled motion” (SM), constructed through the randomization of the starting point position of each stimuli dot, BM coding permits the computation of subtle information inherent to the observed motion condensed in the motion's kinematics (Kozlowski and Cutting, 1977). For example, when observing PLD depicting human movement, volunteers are able to identify gender (Kozlowski and Cutting, 1977), identity (Cutting and Kozlowski, 1977), the ability to dance (Brown et al., 2006), and the subjects' affective state (Pollick et al., 2001, 2002) or facial expressions (Bassili, 1978). However, action recognition is impaired when the volunteers are exposed to static frames of these same PLD, highlighting the relevance of kinematic clues to the coding of biological motion (see a review in Blake and Shiffrar, 2007). Searching for the neural substrates responsible for BM coding, early experiments employing functional magnetic resonance imaging (fMRI) (Grèzes et al., 2001; Grossman and Blake, 2001, 2002; Saygin et al., 2004) and positron emission tomography (PET) (Bonda et al., 1996) posited the superior temporal sulcus (STS) as a putative neural subtract for BM detection. Likewise, EEG studies indicated that the T6 electrode (roughly corresponding to the STS region; see Homan et al., 1987) was implicated in BM coding (Hirai et al., 2003; Jokisch et al., 2005; Krakowski et al., 2011; Saunier et al., 2013).

Studies that corroborate that the visual processing of BM would be closely related to social cognition were reviewed by Pavlova (2012). They suggest that BM could be used as a hallmark to evaluate the social abilities of the individuals, arguing that people with autism, X fragile syndrome and adolescents with periventricular leukomalacia—pathologies associated with the impairment of social cognition—present deficient BM processing. However, in patients with Williams syndrome—a pathology associated with mental retardation, but with a hyper social personality profile—the capability in detecting BM is unimpaired.

Within a plethora of motor repertoires, bipedal posture lies among the most recent mammalian phylogenetic acquisitions (Preuschoff, 2004; Skoyles, 2006). Postural control refers both to body orientation (alignment of body segments in relation to the environment) and body stability or balance (maintenance of body position under internal and external perturbations). Although initially thought of as a subcortical function (Magnus, 1926), postural control has recently been shown to also rely heavily on cortical processing (Deliagina et al., 2006; Jacobs and Horak, 2007). Postural stability is highly influenced by the amount of attention invested in keeping stance (Donker et al., 2007), by previous knowledge of an upcoming postural perturbation (Jacobs et al., 2008) and by the surrounding emotional context (Azevedo et al., 2005; Facchinetti et al., 2006). Therefore, it might rather be considered as a complex cognitive-motor function. Moreover, balance impairment is commonly associated with many supraspinal neurological disorders such as stroke (Spinazzola et al., 2003; Geurst et al., 2005), Parkinson's disease (Horak et al., 1992, 2005) and cerebellar ataxia (Marquer et al., 2014), highlighting the engagement of a distributed brain network in the coding of postural control.

There have been few studies aiming to elucidate the neural processes underlying postural control employing action observation paradigms. Based on the motor simulation theory (Rizzolatti et al., 2001, 2002), where a direct action-perception coupling is proposed, it is expected that the neural networks engaged both during action execution and action observation would be similar (Prinz, 1997; Hommel et al., 2001), and the observation of a situation of postural instability would lead to a postural contagion. In this vein, Thirioux et al. (2009) provided evidence that the observation of a human avatar produces an imitation response in which postural adjustments are made in response to a context of postural instability. This phenomenon was further explored through the use of posturographic measurements (Tia et al., 2011, 2012). Increases in postural sway (mainly in the forward-backward direction) were observed during the presentation of either video sequences of an actress balancing on a gymnastic beam (Tia et al., 2012) or by presenting a PLD obtained from the recordings of a gymnast in a postural instability context (Tia et al., 2011). The concept of postural contagion (i.e., changes in action execution promoted by action observation) was proposed based on these results. Speculation regarding the neural substrates of postural contagion suggests the participation of the mirror neuron system and the existence of higher order processes enrolled in the coding of stance (Slobounov et al., 2000, 2006).

In the present study we investigate whether upright stance would be codified through the activation of the temporal-parietal cortical network, classically enrolled in the coding of biological motion (Blake and Shiffrar, 2007). We hypothesize that the viewing a quiet stance postural sway, contrasted with its scrambled counterpart, would trigger electrophysiological events underpinning BM detection, corresponding to a negative potential at the 150–250 ms time window at temporal-parietal regions, as observed in early reports (Hirai et al., 2003; Jokisch et al., 2005; Krakowski et al., 2011; Saunier et al., 2013). Furthermore, we conjectured that the viewing of PLD depicting high postural instability would also involve the fronto-parietal networks usually enrolled in action observation (Saygin et al., 2004). We address these hypotheses by comparing high-density EEG dynamics yielded by the observation of PLD depicting quiet stable and unstable upright stance.

## Methods

### Stimuli

Four point-light display (PLD) animations, two “biological” and two “scrambled,” were designed for this experiment. These PLD were built from the recording of two male actors, aged 29 and 32 years old, while performing the following tasks: [1] maintenance of the upright stance on a stable surface (quiet posture); and [2] maintenance of the upright stance on a balance board (unstable posture; dimensions of the board: 150 × 60 cm, with a height of 19 cm). The tasks were recorded for 60s using an optoelectronic system (SMART, BTS Bioengineering), with nine infrared cameras positioned in circle, with a sampling rate of 120 Hz. The actors were positioned in the middle of the circle, 3.5 m from the cameras. Fifteen reflexive markers were attached on specific anatomical points on each subject: head (vertex); upper limbs (acromion, lateral epicondyle of the humerus, radial styloid process); hip (anterior superior iliac spine) and lower limbs (lateral epicondyle of the femur, lateral malleolus and fifth metatarsal). Except for the head, the markers were positioned on both sides of the body. Only the frontal PLD view was used.

The two postural situations (quiet and unstable) were executed twice by the two actors, making a total of four PLD biological stimuli (two for each task and actor). For the preparation of the PLD used during the experimental procedure, 3.000 ms length videos were randomly cut taken from the 60 s recorded ones for each condition. These PLD constituted the biological stimuli. For the construction of the scrambled stimuli, two patterns of point-light motion were used (scrambled, non-biological stimuli), with approximately the same visual angle as the biological stimuli (the maximum angles of the stimuli were 9.0° × 11.4°). Body shape was destroyed by randomizing the initial position of the dots so as to prevent the recognition of a human movement pattern. Since the maintenance of original spatial-temporal profiles would lead to a PLD stimulus larger in visual angle than the original biological PLD, mainly in the unstable condition, the average velocity of all the point-lights displayed in each biological stimulus (considering the 3,000 ms time window and the all the points grouped together) was used for each correspondent scrambled stimuli. As the unstable and quiet posture stimuli have distinct velocity profiles, the average speed of the scrambled counterpart was also different: for the quiet posture scrambled PLD it was 0.01 mm.s^−1^, while for the unstable scrambled PLD it was set as 0.31 mm.s^−1^. The horizontal direction (meaning a translational motion) was applied because it was the predominant trajectory displayed by the point-lights in the biological moving stimuli. Thus, the following PLD were presented to the subjects: quiet posture, biological stimulus (QB); unstable posture, biological stimulus (UB); non-biological scrambled stimulus, corresponding to the quiet posture (QS); non-biological scrambled stimulus, corresponding to the unstable posture (US).

### Volunteers

A total of 25 healthy male subjects were evaluated. Thirteen volunteers (age range 19–38 years) participated in the preliminary evaluation of the point-light videos built for this study, and 12 volunteers (age range 20–39 years) were subjected to the EEG data collection. All the subjects had normal or corrected vision, and did not report any neurological, orthopedic or muscular pathology; the volunteers were classified as right-handed, according to the Edinburgh lateral dominance scale (Oldfield, 1971). The volunteers signed an informed consent, after comprehensive information detailing the nature of the study and the protocol to be performed had been given to them. The local ethical research committee approved the present experimental protocol (process number 13481213.4.0000.5257).

### Preliminary evaluation of the stimuli

A preliminary evaluation of subjective perception of the balance/imbalance level for each biological stimulus (QB and UB) was made with a subset of subjects (*N* = 13). We asked the group of volunteers to observe the PLDs and evaluate them with scores ranging from 0 (very balanced) to 10 (very unstable), based on a modified scale as described by Schieppati et al. (1999). The evaluation was made manually during the inter-stimuli fixation cross. The volunteers were positioned seated 60 cm distant from a 19” monitor, in an environment with reduced lighting. Four animations of each of the two conditions were presented using the software Presentation® (Neurobehavioral System). They consisted of two different actors executing each movement twice (quiet posture and unstable posture), totaling 8 events presented randomly, each separated by a fixation cross with the same duration (3,000 ms). The scale assessment was made concomitantly with the fixation period. For the comparison of the instability level perceived between QB vs. UB, we used the nonparametric Wilcoxon test, assuming *p* < 0.05. The results showed a significant difference between QB and UB (*p* = 0.002) in terms of perceived instability, with UB showing higher instability scores than QB: QB score [median (1st–3rd quartile)] of 0 (0–1); UB score of 7 (5–9).

### Experimental procedure

The volunteer sat comfortably in a chair in an environment with reduced lighting. After he was positioned, the experimenter carefully applied the 128 electrode cap to the volunteer's scalp. The instructions given to the volunteer were to remain relaxed in the chair with their eyes open and their gaze on the fixation cross, presented in the center of the LCD monitor (Dell E7909W de 19,” 1152 × 864 pixels, refresh rate of 75 Hz).

**Figure 1:**
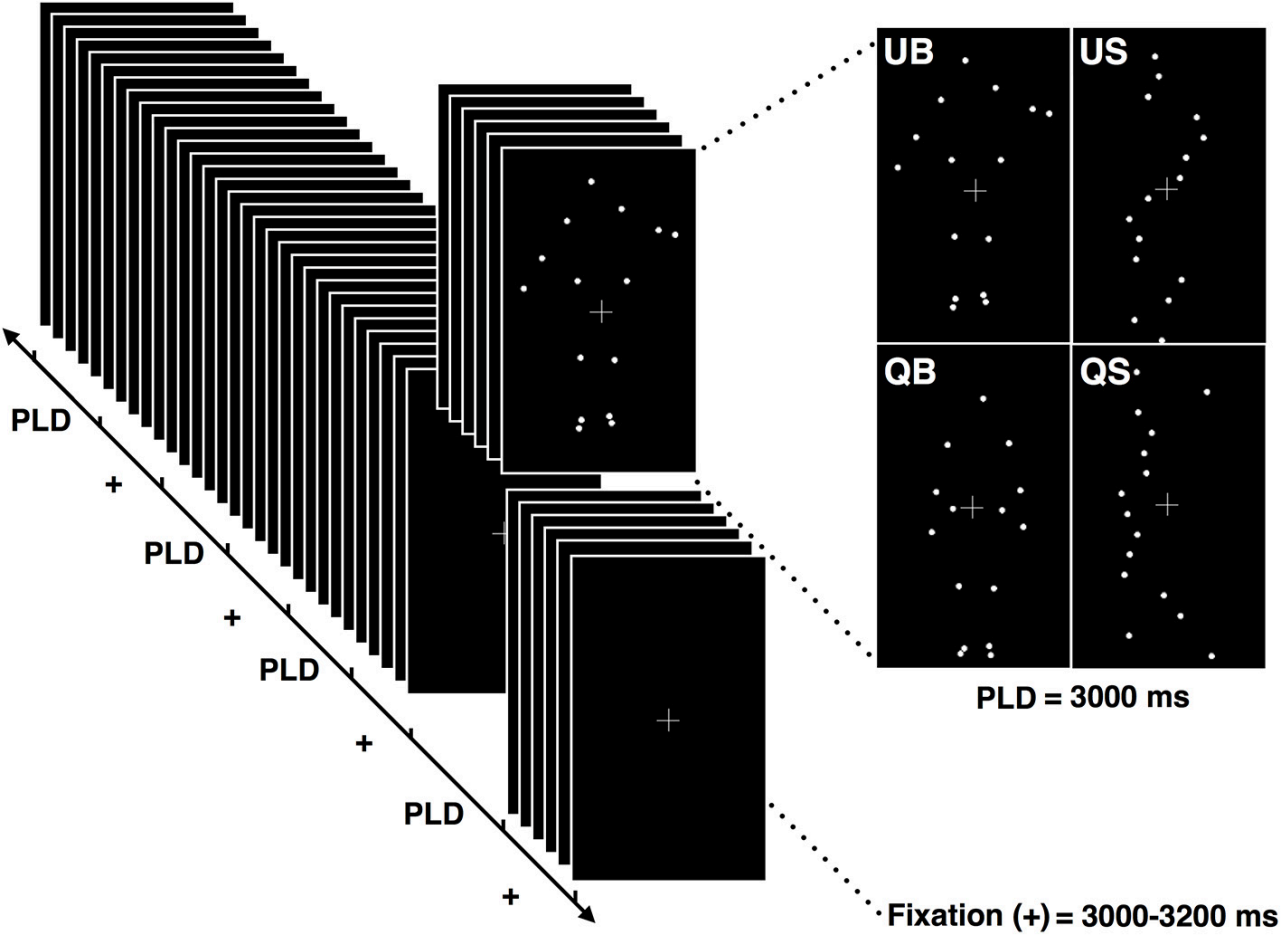
**Schematic illustration of the stimuli presentation protocol**. Each block was composed of 8 point-light displays (PLD) of each of the 4 conditions (QB, quiet biological; QS, quiet scrambled; UB, unstable biological; US, unstable scrambled). Each PLD's condition had a duration of 3.000 ms and was presented randomly, separated by the fixation cross presentation which lasted between 3.000 and 3.200 ms.

The experiment consisted of the observation of a sequence of 8 blocks presented by Presentation® software, with a 5 min' resting interval between block. Each block comprised 32 PLDs (white dots on a black background), being 8 repetitions of each one of the 4 conditions (QB, UB, QS, and US) displayed randomly (Figure 1), totaling 64 presentations of each stimulus. PLD presentation and EEG recordings were synchronized through a digital trigger signal. Each PLD lasted 3,000 ms and was preceded by a fixation cross (interstimulus), presented for a variable time period (between 3,000 and 3,200 ms), to avoid any anticipation or expectancy effects regarding the upcoming animation. The fixation cross was set to help the subjects position their gaze during the experiment, minimizing eye movements, and the last 200 ms of this interstimulus period was also used as a baseline condition. The total duration of the experimental session was approximately 90 min.

After the experimental session, the participants were asked about their subjective perception of the balance/imbalance level presented in each biological stimulus, similarly to that described previously (see “Preliminary evaluation of the stimuli” section). To compare the scores obtained from QB and UB, we used the nonparametric Wilcoxon test, assuming *p* < 0.05.

### Acquisition of the EEG signal

For EEG data recording we used a 128 channel device (Geodesic HidroCel GSN 128 EGI, Electrical Geodesic Inc.) built in a soft cap. The signal was amplified by an optic isolated system of high impedance (200 MΩ) with a converter A/D of 24 bits, and a nominal gain of 20x. The acquisition was performed at a sampling frequency of 500 Hz, and the signal was analogically filtered (Butterworth first order band-pass filter of 0.1–200 Hz; Geodesic EEG System 300, Electrical Geodesic Inc.). The impedance of the electrodes was maintained below 60 kΩ for all the recordings. The electrode positioned on the vertex (Cz) was used as a reference during the acquisition.

### Pre-processing of the EEG signal

Firstly, the recorded signal was re-referenced from Cz to the average reference using NetStation software (Electrical Geodesic Inc.). Following this procedure, the data was analyzed offline using EEGLab in MatLab's environment (Math Works, Version 2011a). The raw data was segmented in events of 4,000 ms, discarding the first 1,000 ms of the fixation cross and the last 1,000 ms of the PLD of each event. This segment duration was chosen considering a window of interest of the first 1,000 ms after the stimuli onset (see also Hirai et al., 2003; Jokisch et al., 2005; Krakowski et al., 2011; Saunier et al., 2013). A proportional segment (1,000 ms) of fixation cross period was employed to calculate the baseline. Next, a baseline correction was applied using the last 200 ms of the fixation cross signal period. After that, the signal was digitally filtered in a 0.5–50 Hz band (FIR filter) and a notch filter between 59 and 61 Hz was applied to reduce electrical interferences from the power grid.

After a visual inspection (made over electrode 21, relative to the channel FP1 in the 10–20 international system) to exclude large fluctuations in the signal, as well as eye-blinks and artifacts, the final data consisted of a minimum of 70% of good trials per volunteer.

### ERP analyses

The following analyses were run in R software environment. To investigate the cerebral dynamics during the observation of postural adjustments, we applied a statistical approach similar to that of Foxe and Simpson (2002) and Krakowski et al. (2011). Point-by-point paired-T tests (considering the whole 2,000 ms trial) were calculated between EEG time series acquired in each condition (quiet vs. unstable) and in both patterns of point-light motion used (biological and scrambled stimuli).

Differences were considered significant only when the following spatial-temporal condition was satisfied: let *T*_*k*_(*t*) be the *T-*score of electrode *k* at time *t*, and let *W*_*k*_(*t*) be this value averaged over 21 consecutive time points (42 ms), 10 before and 10 after *t* (Equation 1),

(1) $$\begin{array}{r} W_{k}\left(t\right)=\frac{1}{21}\sum_{s=-10}^{10}T_{k}\left(t+s\right). \end{array}$$

and let $W_{k}^{neig}$(*t*) be the following average over the three nearest neighbors of electrode *k* (equation 2).

(2) $$\begin{array}{r} W_{k}^{neig}\left(t\right)=\frac{1}{3}\underset{l,m,n\in \theta_{5}}{\max}\left\{\left|W_{l}\left(t\right)|+|W_{m}\left(t\right)|+|W_{n}\left(t\right)\right|\right\}, \end{array}$$

with *l*≠*m*≠*n*, and where θ_5_ is the set of the five nearest neighbors of electrode *k*. (Equation 2)

**Figure 2:**
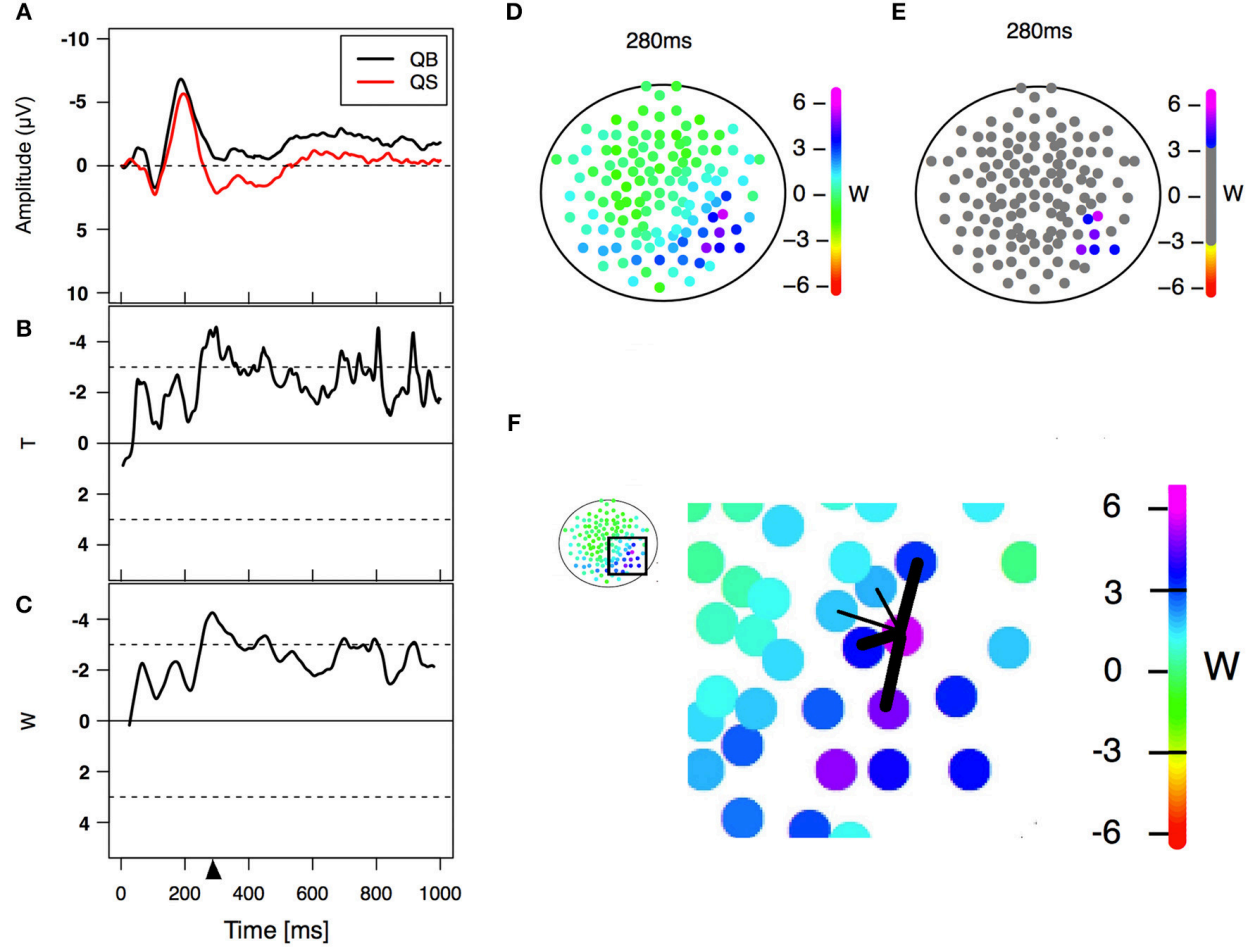
**Statistical analysis. (A)** Example of an event-related potential obtained in two different PLD conditions in a particular electrode. **(B)** Paired *T*-statistics as a function of time for comparing both PLD conditions on the same electrode. **(C)** W-statistics as a function of time, computed as a time average of the *T*-statistics over a time windows of 21 points/42 ms (Equation 1). The triangle refers to a specific time point (280 ms in this case) corresponding to the center of the window of 21 points/42 ms width (left lower panel) used for statistical analysis. **(D)** W-statistics plotted in a topological distribution map for time equal 280 ms. Each colored electrode depicts its particular *W*-value. **(E)** Electrodes are considered to have a significant difference between conditions if |*W*|>3 and a similar behavior is observed in their spatial neighborhood (Equation 2). **(F)** Scheme showing an example of the spatial neighbors' criterion. The five nearest neighbors are selected and the average of the three largest |*W*| values (thick links) is computed. If the values *W*^*neig*^ and |*W*| are greater than 3 then the difference is considered significant for that electrode. This procedure is done for all 128 electrodes obtaining at the end the result shown in **(E)**.

Differences were considered significant for electrode *k* at time *t* if |*W*_*k*_(*t*)|>3 and $W_{k}^{neig}$(*t*)>3. Therefore, the activity expressed in electrode *k* was only considered as significant if this electrode *k* has both large differences between conditions (|*W*_*k*_(*t*)|>3) and a similar behavior was found in its closest neighbors ($W_{k}^{neig}$(*t*)>3). This last condition in relation to its neighbor permitted an electrode to be identified as significant only if one of the five nearest neighbors had a very large difference [such that the average value $W_{k}^{neig}$(*t*) is greater than 3] or if at least three of them present large (|*W*_*i*_(*t*)|>3) between condition differences. A schematic illustration of this procedure is presented in Figure 2.

**Figure 3:**
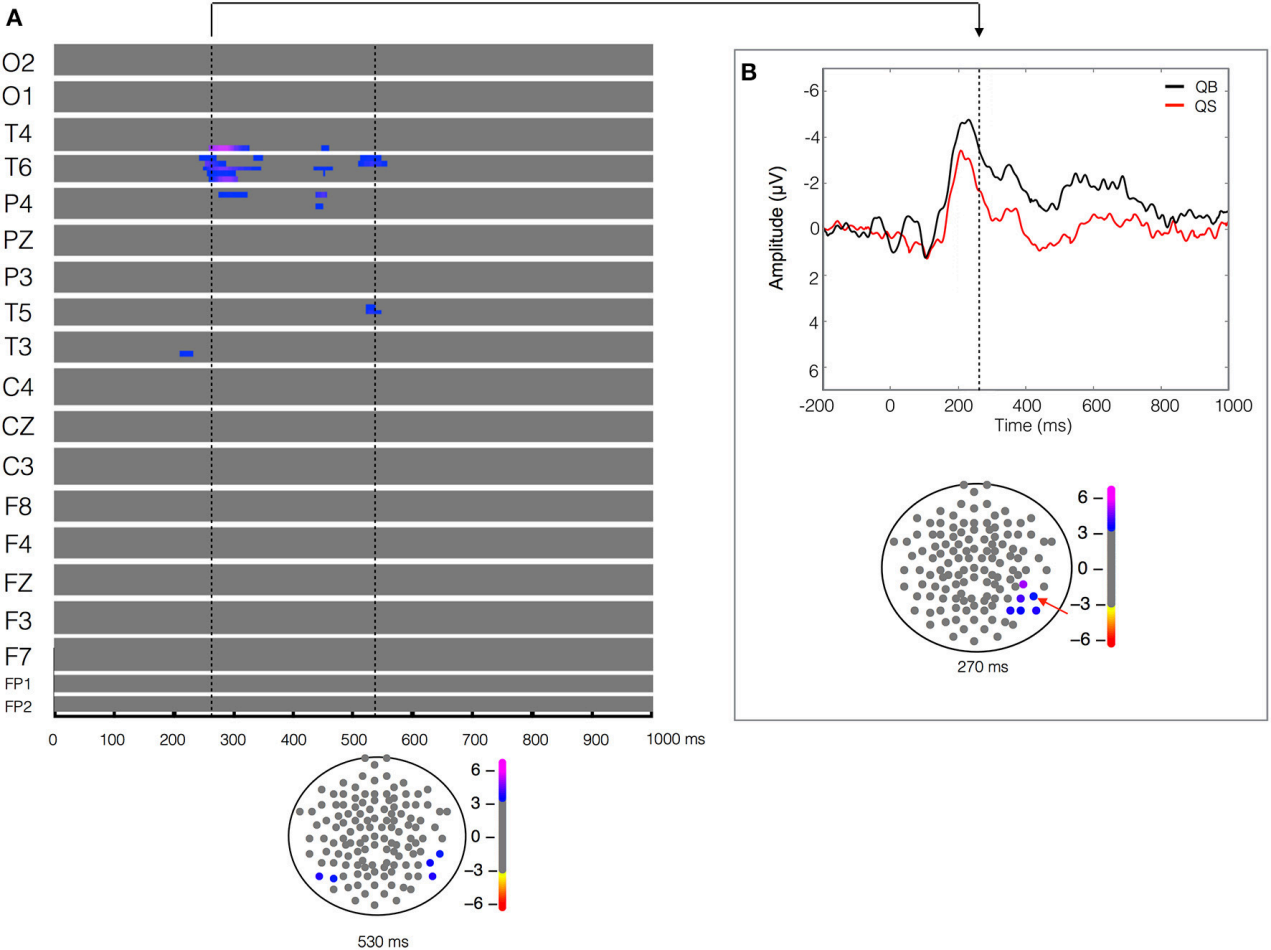
**Quiet biological (QB) vs. Quiet scrambled (QS)**. Plot of *W*-values for QB vs. QS contrast (upper panel in **A**), highlighting events at the 270 and 550 ms time points (vertical lines). Topological distribution of the differences is plotted in a 21-point temporal window centered at 270 ms time point in the bottom panel of **(B)**. The corresponding event-related potentials obtained from temporal-parietal electrodes (inset red arrows) in the correspondent time point are presented in the upper panel.

**Figure 4:**
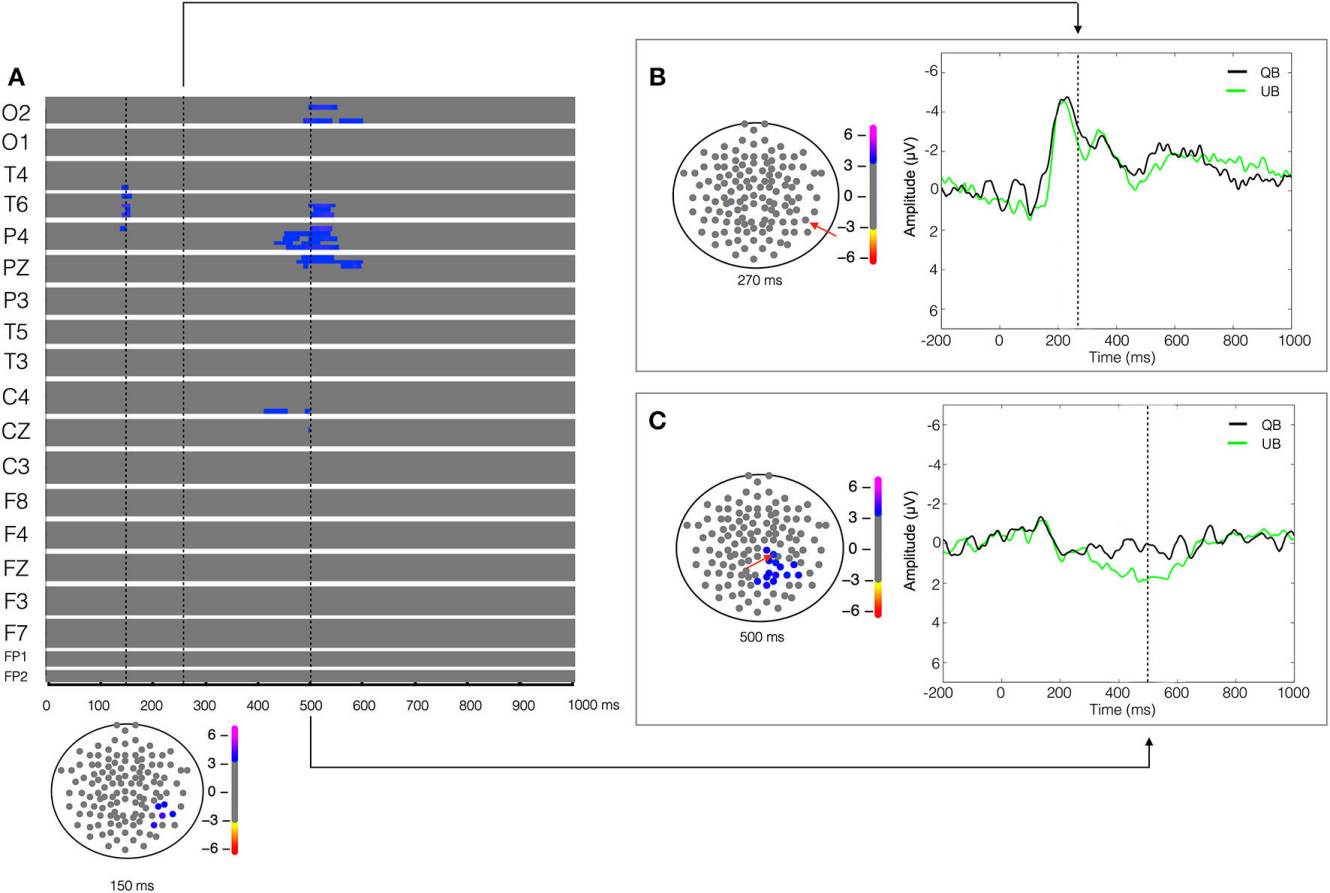
**Unstable biological (UB) vs. Quiet biological (QB) contrast**. Plot of *W*-values for UB vs. QB contrast (upper panel in **A**), highlighting events at the 150, 270, and 500 ms time points (vertical lines). Topological distribution of the differences is plotted in a 21-point temporal window centered at each time point in the bottom panel in **(A)** and in the left panels in **(B,C)**, respectively. The corresponding event-related potentials obtained from temporal **(B)** and parietal **(C)** electrodes (inset red arrows) in the correspondent time point are presented in the right panels.

The rationality behind the criterion was the following: as we wanted to eliminate spurious differences due to multiple testing, the way we tackled this problem was by requiring that the differences lasted for a long period (average over 42 ms) and that they were sufficiently important for a group of neighboring electrodes to express this difference. The co-occurrence of both conditions generates a robust criterion for identifying the two different conditions, as shown in Figures 3, 4. The lack of significant differences between EEG recordings acquired during the inter-stimulus (fixation cross) interval preceding the presentation of biological and scrambled stimulus (data not shown) confirms the strength of the proposed method.

## Results

### Evaluation of the stimuli

When we compared the instability level between QB vs. UB perceived by the volunteers, a significant difference between a QB score [median (1st–3rd quartile)] of 0 (0–1) and a UB score of 6 [(6–9), *p* = 0.017] was found. These results suggest that the volunteers were capable of correctly identifying the different PLDs as depicting situations of QB or UB. Complementary to this evaluation, the volunteers indicated how easily they identified human figures in the PLD presented during the experiment, using a scale ranging from 1 (easy to perceive) to 5 (hard to perceive). All the subjects reported scores between 1 and 2, indicating that they were able to easily identify a human figure in both PLD.

### Biological components in quiet and unstable stance

The ERPs recorded during the visualization of the two stimuli (biological vs. scrambled) in each of the two postural conditions (quiet and unstable posture) were compared. The result of the paired-T test between the quiet biological and quiet scrambled stimulus (QB vs. QS) showed a significant difference (*W* = 5.14, *p* < 9 × 10^−5^) in the 200–300 ms time window, more pronounced in the right temporal-parietal region (T4, T6 and P4 electrodes) and less pronounced but also evident in a left temporal (T3) electrode (Figure 3). As shown in Figure 3B, this statistical difference corresponded to a higher and longer lasting ERP negativity in the QB condition. This ERP signal has a classic biological motion detection profile, also previously shown for other types of motion (Hirai et al., 2003; Jokisch et al., 2005; Krakowski et al., 2011; Saunier et al., 2013). This difference with the QS condition was also clearly evident but less pronounced at 400–550 ms after the stimulus onset on the same electrodes (T4, T6, and P4), and included the left temporal electrode (T5). No other relevant statistical results were observed.

In respect of the contrast between the unstable biological stimulus (UB) and its scrambled counterpart (US), between-condition differences were expressed in the 500–620 ms time window by a greater positivity for UB in the central-parietal regions (P3, PZ, C4, and mainly CZ), while a greater negativity appeared for UB in a frontal electrode (F8). No other relevant statistical results were observed (Supplementary Figure 1A).

### Comparison of two different levels of postural imbalance stance

Aiming to understand the cerebral dynamics related to the codification of the degree of postural instability, we contrasted the two biological conditions (UB vs. QB). The result of the paired-T test showed an early difference (around 150 ms) in the right temporal-parietal regions (T4, T6, and P4), reflected by a greater positivity (*W* = 4.16, *p* < 4 × 10^−4^) in the EEG signal during the UB condition (Figure 4). As expected, no significant between-condition activity was found in the temporal-parietal electrodes in the biological motion detection window (170–250 ms), indicating that both stimuli were labeled similarly as biological motion (Figure 4B). Furthermore, in the 400–600 ms temporal window a large between condition difference, lateralized in the right hemisphere and characterized by a higher positivity (*W* = 3.92, *p* < 6 × 10^−4^) in the UB condition was found in the occipital (O2), temporal (T6), parietal (P4 and PZ), and central electrodes (C4) (Figure 4C). No other significant differences were observed.

Finally, in order to verify that the previous result was not due to low-level visual information differences in the PLD (as their velocity or spatial profile), we contrasted the two scrambled stimuli (US and QS). The result of the paired-T test showed that all the between-condition differences found when we contrasted the biological stimuli (UB vs. QB) disappeared when we compared their scrambled counterparts (US vs. QS), indicating that these EEG differences relied on the BM properties of the stimuli, and not on their low-level visual characteristics. Besides, only a late activity around 880–980 ms after the stimulus onset in the left occipital (O1) and left temporal-parietal (T5, PZ and P3) electrodes, expressed by a more pronounced positivity in the US condition (Supplementary Figure 1B) was observed.

## Discussion

In the present study we compared high-density EEG dynamics yielded by the observation of PLD depicting quiet stable and unstable upright stance to investigate whether upright stance would be codified through the activation of the temporal-parietal cortical network classically enrolled in the coding of biological motion (Blake and Shiffrar, 2007). Through the paradigm of biological motion we aimed at the underlying perceptual-cognitive features associated with postural contagion and postural control. Our approach was based on the concept of a direct action-perception coupling, as proposed by Rizzolatti et al. (2001, 2002). Within this framework, it is proposed that neural networks are similarly engaged both during action execution and action observation (Prinz, 1997; Hommel et al., 2001). In this vein, there is evidence of changes in postural control when subjects either observe a human avatar (Slobounov et al., 2000, 2006; Tia et al., 2011, 2012) or during motor imagery of postural-related movements (Rodrigues et al., 2010; Lemos et al., 2014). Our main results were that observing a person sustaining a quiet stance posture, as compared with its scrambled counterpart, leads primarily to the recruitment of the temporal and parietal regions of the right hemisphere. In addition, postural instability was coded in the central, but also in the parietal and temporal regions, slightly lateralized on the right hemisphere. These results are discussed in detail below.

### Biological vs. scrambled contrasts reveal the biological content of postural stance

Contrasting the stable conditions (QB vs. QS) revealed the presence of a negative peak, detected at a latency ranging between 200 and 300 ms after the stimuli onset over the temporal-parietal region, mainly in the right hemisphere (Figure 3). This is the classical between-condition difference (biological vs. scrambled) previously found during the observation of PLDs depicting several types of human movements (Hirai et al., 2003; Jokisch et al., 2005; Krakowski et al., 2011; Saunier et al., 2013). Thus, our data suggest that the brain codes quiet stance (QB) as a biological movement entity.

The superior temporal sulcus (STS) is classically associated with the perception of biological motion (Bonda et al., 1996; Grossman and Blake, 2001, 2002; Hirai et al., 2003; Puce and Perrett, 2003; Jokisch et al., 2005; Krakowski et al., 2011; Saunier et al., 2013) and is also recognized as an integrative area of inputs from the ventral and dorsal streams (Giese and Poggio, 2003), respectively, the form and motion visual pathways. Peuskens et al. (2005) showed higher STS activity in response to the presentation of BM than to a simple 3D rotation of a frozen BM frame, demonstrating the importance of kinematic information for the STS activation. In the same vein, Vangeneugden et al. (2014) associated the posterior STS with the detection of motion patterns, whereas higher visual areas such as the extrastriate body area (EBA) were more important in the discrimination of body form information. It is important to acknowledge that the subtlety of the stimulus motion in the quiet stance would probably not be perceived from completely static dots. In fact, when Buzzell et al. (2013) compared static frozen frames of BM and SM, the EEG temporal-parietal N1 peak that they had found between classical BM vs. SM disappeared. Applied to the present results, the engagement of the temporal-parietal regions during the observation of a QB condition is possibly due to a form-from-motion process, suggesting that even the lowest level of joint motion can be sufficient to transform static meaningless dots into coherent postural motion, as previously shown through behavioral experiments (Johansson, 1973, 1976).

The right temporal electrode T6 presented a significant between-condition difference (QB vs. QS) for almost more 300 ms, corroborating the right temporal EEG activity found by Saunier et al. (2013) when subjects observed PLDs depicting biological locomotion as compared to its scrambled counterpart. Using a similar paradigm, Krakowski et al. (2011) interpreted these later differences as “cognitive processes involved in decoding the meaning of the activity displayed by the motion stimulus” (p. 381), possibly related with the computation of the stimuli attentional load. Applied to the present results, this attentional load could correspond to extracting the meaning of this intransitive motion (i.e., the maintenance of orthostatic posture). On the other hand, Sitnikova et al. (2003, 2008) also found a late ERP component (500–800 ms) during the observation of reaching movements which were incongruent with the action goal compared to those that were congruent.

We expected to find the N1 peak difference found for the BM vs. SM contrast in the temporal-parietal regions also for the UB vs. US contrast. However, both the UB and the US conditions produced an ERP in the same temporal-parietal regions as the quiet stance contrast, resulting in an absence of difference between UB and US. We believe that this unusual result might be due to the nature of the control stimulus used in the unstable condition. The introduction of translational moving dots in the scrambled motion condition (US) might have produced a more identifiable pattern than those evoked by typical scrambled stimuli. Indeed, some volunteers reported seeing a rocket or a rotating DNA form. Thus, the expected between-condition difference could be less pronounced than that obtained by comparing the usual BM vs. SM. Indeed, Peuskens et al. (2005) have previously shown that translational dots moving in a same direction were sufficient to promote an increase in the activity of the STS. It is worth noting however, that after the EEG experiment, the volunteers unanimously considered very easy to detect human motion in the biological conditions (UB and QB). Despite the possible problems deriving from the manipulation of the spatial-temporal properties of the scrambled stimuli, our priority was to maintain the visual angle comparable between the biological and their scrambled counterparts. Besides, manipulations of the classical profile of scrambled stimuli had been applied before by Buzzell et al. (2013) and White et al. (2014), for instance.

For the UB vs. US contrast, we found a later (500–620 ms) between-condition difference expressed by a central-parietal positivity (P3, PZ, C4, and CZ) and a right frontal (F8) negativity, both more pronounced for the UB condition. This result seems closely related with those reported by White et al. (2014), who found a medial parietal positivity (MPP) and a ventral anterior negativity (VAN) when comparing biological motion with its scrambled counterpart. The authors argued that these later changes in potential indicated a more complex cognitive processing, inherent to the codification of biological motion. This can explain why such MPP/VAN appear only for the UB vs. US contrast, since an unstable situation suggests a more difficult and threatening situation than a quiet one, as discussed in the following section.

### Postural instability is processed in a later phase of BM codification

From an examination of the contrasts found between different postural contexts, we aimed to evaluate the cerebral dynamics associated with the codification of postural instability. In our UB vs. QB contrast, the absence of the temporal-parietal between-condition difference in the 170–250 ms time window—a typical marker for the detection of BM in PLD paradigms depicting human motion (Hirai et al., 2003; Jokisch et al., 2005; Krakowski et al., 2011; Saunier et al., 2013)—strongly argues that both conditions are recognized (QB and UB) as biological motion (Figure 4), reinforcing the arguments of the previous section. Moreover, we found an earlier between-condition difference (around 150 ms) in the right temporal-parietal regions (T4, T6, and P4), reflected by a greater positivity for the UB condition. This finding could be a consequence of the emotional load of the postural instability context resulting in an increased feeling of threat, intrinsic to a situation where the difficulty of maintaining an orthostatic posture is set by a context of high instability on the ground. Consistent with this hypothesis, in a recent study using magnetoencephalography (MEG) Meereen et al. (2016) showed early (80–110 ms) right parietal activation in response to pictures of fearful postural bodies, in comparison to neutral ones, pointing to an early emotional processing whenever a threatening nuance is detected in the observed stimulus. In another line of evidence, upright stance has proven to be highly susceptible to emotional contexts induced by picture viewing (Azevedo et al., 2005; Facchinetti et al., 2006). Employing an event related fMRI design, Vuilleumier et al. (2001) reported the early activation of the left amygdala upon threat-related facial expressions, irrespectively if they were attended or not. Later on, employing a very similar paradigm, Vuilleumier et al. (2004) showed a reduced activity in the fusiform and occipital cortex in patients with medial temporal lobe sclerosis. Such reduction was proportional to the amygdala damage, thus indicating that the amygdala acts as a main drive of the fusiform/occipital regions in visually driven fearful contexts.

Interestingly, when considering the UB vs. QB contrast, a lateralized right hemisphere activity was found during the 400–600 ms time window in the occipital (O2), temporal (T4 and T6), parietal (P4 and PZ), and central electrodes (C4 and CZ). We suggest that such differences are directly involved in the coding of postural instability, similarly to the “neural detector” of instability concept proposed by Slobounov et al. (2000, 2006). Employing EEG and neuroimaging, Slobounov and his team suggested a fronto-centro-parietal “neural detector” for postural instability, activated when subjects were instructed to visually recognize non-stable postures of an animated avatar (Slobounov et al., 2000). The engagement of a similar network preceding the reaching of limits-of-stability boundaries when subjects are in a real postural instability context reinforces this concept (Slobounov et al., 2005). Furthermore, being described as the core of the action-perception network (Iacoboni and Dapretto, 2006; Blake and Shiffrar, 2007), the central-parietal regions would be highly engaged in action coding. Previous studies have already pointed to a late modulation of cerebral dynamics during the observation of PLD in an attended condition (Krakowski et al., 2011). In fact, in this high-density investigation of biological motion detection, a significant central-parietal positivity was observed at a time window above 400 ms.

Finally, in our last contrast, US vs. QS, our main objective was to verify that the cortical activity was related to postural instability and not the result of differences in the velocity and trajectory area of the moving dots between the UB and QB situations. If this was so, we would expect to obtain a similar profile when comparing the biological (UB vs. QB) and the scrambled (US vs. QS) conditions, which was not the case (see Supplementary Figure 1B). The completely different patterns of activity in these two contrasts support the idea that our results obtained from comparing the biological conditions, previously discussed, are due to significant differences in the processing of disparate postural adjustment situations.

### Predominance of right-lateralized response to postural-related PLD

In the present study we found a predominant activation in the right hemisphere during the observation of PLD depicting different postural contexts. The right temporal sulcus has already been associated to the detection of BM in experiments using EEG (Hirai et al., 2003; Jokisch et al., 2005; Krakowski et al., 2011; Buzzell et al., 2013; Saunier et al., 2013; White et al., 2014), PET (Bonda et al., 1996), and fMRI (Peuskens et al., 2005). Other fMRI investigations revealed however a bilateral activation of the posterior STS during the PLD observation (Grossman and Blake, 2001, 2002). Likewise, employing a behavioral experiment with stroke patients, Saygin (2007), showed that the recognition of BM was impaired when the volunteers had lesions either on the right or the left STS. Future studies may shed further light on the nature an extent of BM coding lateralization.

The codification of postural instability was herein accompanied by a wide change in cerebral electrical activity, mainly in the temporal and central areas of the right hemisphere (see the Results section, Figures 4A,C). Moreover, it seems plausible to speculate about a right hemisphere-centered system for “balance coding,” given the evidence about the importance of the right cerebral regions in postural control. From the clinical point of view, evidence points toward a higher incidence of Pusher syndrome (in which the patient actively pushes himself toward the contralateral side of their brain lesion) in right-hemisphere injured patients (Karnath et al., 2000; Karnath, 2007). In addition, postural disorders are commonly observed in patients presenting altered structural and functional lesions in the right hemisphere (Spinazzola et al., 2003). Both conditions seem to be related to dysfunctional postural representations or postural schema, crucial for proper organization of postural adjustments in response to internal and external perturbations (Massion, 1992; Massion et al., 2004). Despite this set of evidences, a bilateral system for postural instability detection has been proposed (Slobounov et al., 2000, 2006; Taube et al., 2015). Whether postural instability is detected by a bilateral system or not, our and previous findings are all consistent with the proposition of a balance coding system in the brain. Given the quite specific patterns of EEG dynamics found during postural context observation, it seems tempting to propose that the right hemisphere bears a crucial role in balance control.

## Limitations and concerns

Some caution appears warranted when interpreting neural correlates in order to conclude how encoding takes place in the brain. Lesion or brain stimulation studies testing both postural sway and biological motion perception may allow confirming whether postural sway is truly encoded in the biological motion network. Likewise, the generalization of the biological motion stimuli from a small stimuli sample might be considered as an issue. To the best of our knowledge, this problem has not yet been addressed systematically. Thus, it might be desirable in future studies to evaluate systematically the effects of PLD stimuli variability.

## Conclusions and perspectives

Our results suggest that the brain codes postural adjustments as biological motion, activating similar temporal-parietal networks previously shown for other actions depicted in PLD. Furthermore, postural instability seems to be computed in a central-temporal-parietal network slightly lateralized in the right hemisphere. To disentangle the function of these nodes and their role in coding of postural control, further explorations of this cortical network and its relation with other brain structures is warranted. For example, the cerebellum has been related to a myriad of motor and cognitive functions (e.g., Vožeh, 2016), and previous investigations have linked its activity both with biological motion processing (for example, see Sokolov et al., 2010, 2012) and control of postural adjustments (Horak and Diener, 1994; Lalonde and Strazielle, 2007). Concerning postural instability coding, the findings of Slobounov et al. (2006) of a bilateral activation of cerebellar regions during the active recognition of unstable posture highlights its involvement in perceptual-cognitive-motor processes underlying postural control. Future studies shall unravel the role of this and other brain regions in detecting and coding postural instability.

## Ethics statement

This study was carried out in accordance with the recommendations of Comitê de Ética em Pesquisa from the Hospital Universitário Clementino Fraga Filho, with written informed consent from all subjects. All subjects gave written informed consent in accordance with the Declaration of Helsinki. The protocol was approved by the local committee, process number 13481213.4.0000.5257.

## Author contributions

TL, GS, TP, and CV designed the experiment, EM and TL conducted the experiment, DF and EM analyzed the data, EM, TL, GS, TP, DF, and CV wrote the paper. All authors reviewed the manuscript.

### Conflict of interest statement

The authors declare that the research was conducted in the absence of any commercial or financial relationships that could be construed as a potential conflict of interest.

## Supplementary material

Supplementary Figure 1 **(Upper panel A)** Unstable biological (UB) vs. Unstable scrambled (US)—Point-to-point paired *T*-test comparing the two conditions (UB vs. US) shows a higher positivity for UB in the central-parietal electrodes, while a pronounced negativity is verified in the right temporal and bilateral frontal regions (450–650 ms time window). (Upper panel **B**) Unstable Scrambled (US) vs. Quiet scrambled (QS)—Point-to-point paired *T*-test comparing the two conditions (US vs. QS) shows a significant between-condition only after 850 ms of the stimuli onset. (Bottom panels, **A,B**) Topological distribution of these differences in a 21-point temporal window centered in 600 ms **(A)** and 900 ms **(B)**.
