# Supplementary figures and images for: Cerebral Dynamics during the Observation of Point-Light Displays Depicting Postural Adjustments
Source: Front Hum Neurosci. 2017 May 8;11:217. doi: 10.3389/fnhum.2017.00217 (PMC5420589; doi:10.3389/fnhum.2017.00217)

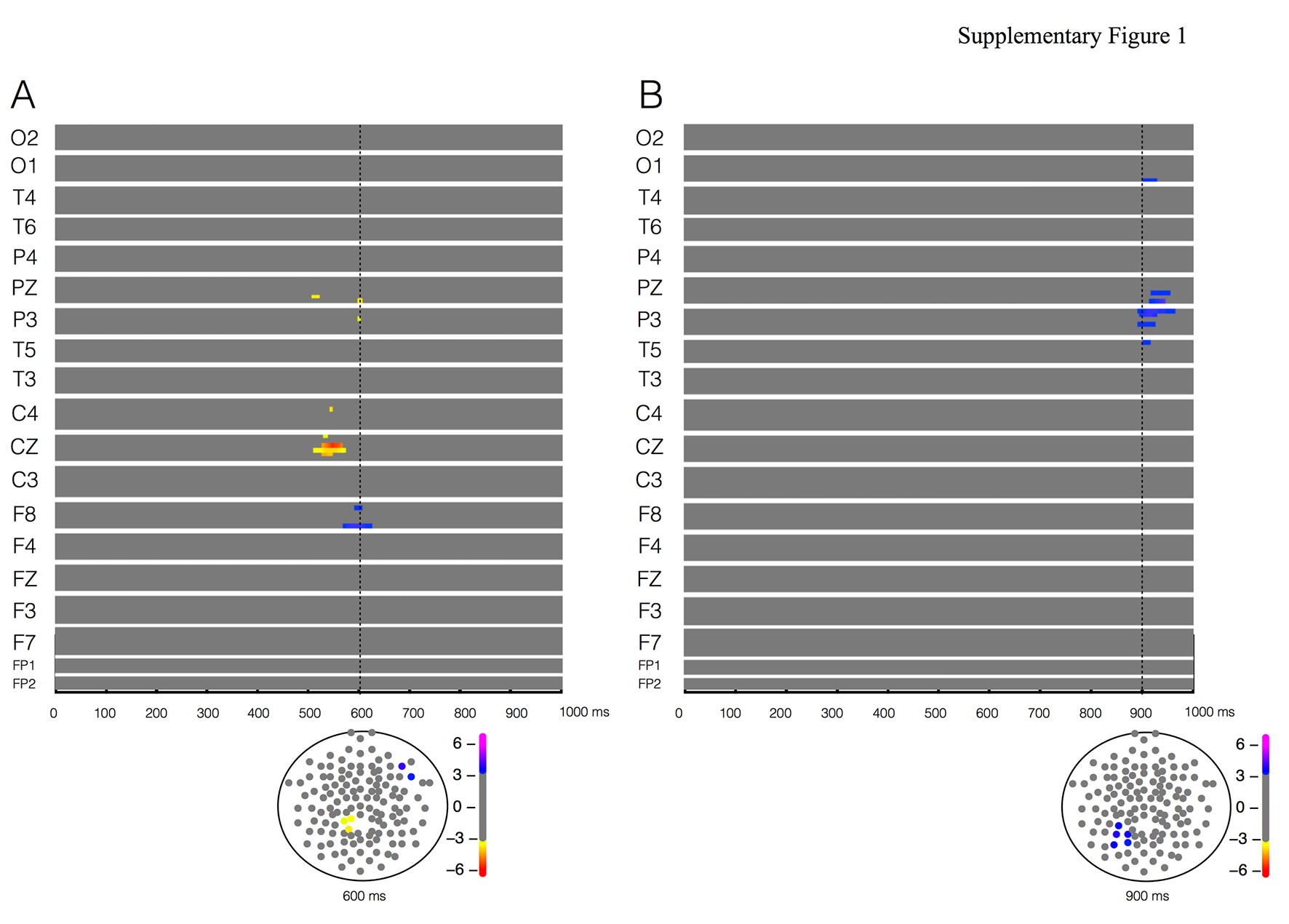

Supplement: Supplementary Figure 1 — (Upper panel A) Unstable biological (UB) vs. Unstable scrambled (US)—Point-to-point paired T-test comparing the two conditions (UB vs. US) shows a higher positivity for UB in the central-parietal electrodes, while a pronounced negativity is verified in the right temporal and bilateral frontal regions (450–650 ms time window). (Upper panel B) Unstable Scrambled (US) vs. Quiet scrambled (QS)—Point-to-point paired T-test comparing the two conditions (US vs. QS) shows a significant between-condition only after 850 ms of the stimuli onset. (Bottom panels, A,B) Topological distribution of these differences in a 21-point temporal window centered in 600 ms (A) and 900 ms (B). [file Image1.TIFF]
